# Macroscopical and histological characterization of the perineal membrane and deep perineal pouch

**DOI:** 10.1111/joa.70057

**Published:** 2025-10-22

**Authors:** Morten Kampelmann, Carina Grindley, Christian Mühlfeld, Andreas Schmiedl

**Affiliations:** ^1^ Institute of Functional and Applied Anatomy Hannover Medical School Hannover Germany

**Keywords:** deep transverse perineal muscle, morphometry, perineal membrane

## Abstract

The existence of a striated deep transverse perineal (DTP) muscle remains an ongoing debate. We hypothesize that the DTP is macroscopically and microscopically detectable as a definable striated muscle belly in elderly males, but not in elderly female donors used in the dissection course. Investigations were performed on embalmed human donors of both sexes (*n* = 10, mean age at death: 82 years). We dissected the ischioanal fossa. After removing all the loose fat and connective tissues, including the arteries and nerves, we exposed the compact transversally running plate‐shaped tissue stretched between the ischiopubic rami. Additionally, we removed the ischiocavernosus and bulbospongiosus muscles in the superficial perineal pouch along with the external genitals. The entire tissue plate located in the deep perineal pouch was then detached from the surrounding bony structures and dissected. To verify the musculature and its striation, we analyzed the tissue stereologically using light microscopy. At 40 μm intervals, 4‐μm‐thick serial sections were made perpendicular to the surface of the dissected tissue block. We determined the volume densities of the connective tissue, smooth muscle cells, and striated muscle fibers in the selected sections relative to the entire tissue in the deep perineal pouch. A relatively thick tissue plate (diameter: 4–5 mm) located superior to a fascia‐like structure, the perineal membrane, was observed running transversally to the ischiopubic rami. Macroscopically, a clearly visible transverse running muscle was not observed in both sexes. Histologically, serial sections showed that the samples mainly consisted of connective tissue. In both sexes, the volume densities of the connective tissue were approximately 80%. In females, the volume density of the muscle cells was approximately 20%. In males, about 10% of the tissue was smooth, whereas just over 10% was striated muscle. However, the standard deviation between the donors was remarkable. In females, the proportion of the striated muscle cells was less than 1%. With increasing age, the volume density of the connective tissue increased, whereas those of the smooth muscle cells and/or striated muscle fibers decreased, independent of sex. There was no evidence of the existence of a clearly definable striated DTP muscle, not only in females but also in males. Muscle cells decreased with increasing age.

## Graphical abstract

The existence of the deep transverse perineal muscle remains controversial. While the macroscopic appearance of the tissue in the deep perineal pouch differs between men and women, the differences in the histological composition of this fibromuscular plate is still unclear. Our results showed that both sexes have a muscle volume density of almost 20%. However, only smooth muscle cells were detected in females, whereas a significant proportion of striated muscle fibers was also present in males.

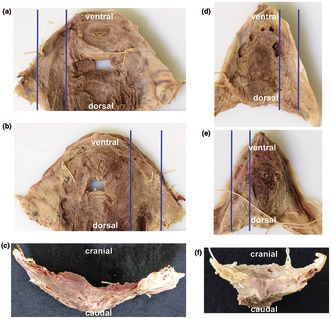

## 1 INTRODUCTION

**FIGURE 1:**
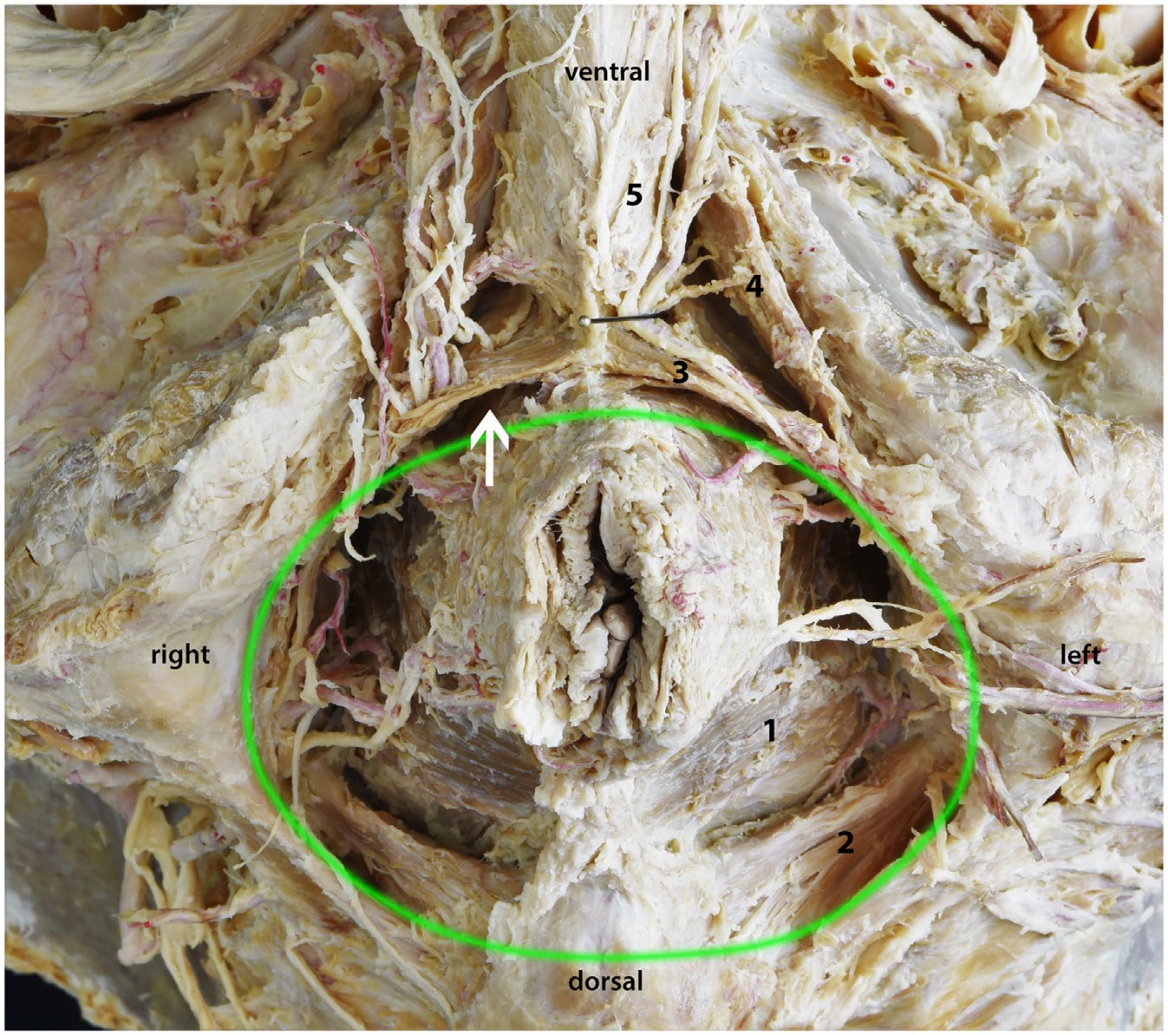
Topography of the perineal region in a male. Dorsally, the anal region is formed by the ischioanal fossa (green circle). On the ventral side is the urogenital region with the deep and superficial perineal pouches. The loose connective tissue extends over (cranial) the deep perineal pouch (arrow). (1) levator ani muscle, (2) coccygeus muscle, (3) perineal membrane/deep transverse perineal muscle, (4) ischiocavernosus muscle, and (5) bulbospongiosus muscle.

The perineal region between the os ischii, pubic symphysis, os coccygis, and pubic rami can be divided into anterior and posterior sections. Anteriorly lies the urogenital region with the superficial and deep perineal pouches and urogenital hiatus. Posteriorly is the anal hiatus and ischioanal fossa. The ischioanal fossa is a pyramid‐shaped space in the posterior perineal region outside the pelvic floor (Figure 1). It is a fat‐filled space in which vessels and nerves run to the perineal region. The ischioanal fossa is bounded medially by the external sphincter and the levator muscles, laterally by the internal obturator muscle and ischial tuberosity, dorsally by the gluteus maximus muscle, and ventrally (cranially) by the posterior edge of the deep perineal pouch with the perineal membrane (Figure 1).

The pelvic cavity is bounded caudally by the pelvic diaphragm, which consists of the levator ani and coccygeus muscles. Clinicians use the term urogenital diaphragm very frequently to describe the pelvic floor. They describe the urogenital diaphragm as a trilaminar structure composed of a striated muscle, called the deep transverse perineal (DTP) muscle, with inferior and superior fascial coverings located caudally between the ischium and the pubic symphysis, to separate the perineal pouch. It is crossed by the urethra and the vagina (Stein & DeLancey, 2008). It was assumed that the urogenital diaphragm is also responsible for continence because fibers of the DTP muscle partly or completely enclose the urethra (Dorschner et al., 2001). However, till date, the presence of the DTP is controversially discussed in the literature (Dorschner et al., 1999, 2001; Fritsch et al., 2004; Kaye et al., 1997; Kureel et al., 2011; Mirilas & Skandalakis, 2004; Nakajima et al., 2007; Roch et al., 2021; Thiele et al., 1997). A separate extrinsic striated sphincter muscle was also discussed (Dorschner et al., 1999, 2001). Henle (1873) described the existence of an upper and lower aponeurosis in men, running below the prostate gland and above the urethral corpus cavernosum between both ischiopubic rami (Henle, 1873). It was assumed that the fasciae enveloped the DTP muscle, which was originally described in the year 1864 by Luschka (Krosigk, 2012). However, the newborn, infant, and male transversal and frontal sections of the pelvic floor exhibited no continuous DTP muscle. A comparison of the pelvic floor and lower urogenital tract anatomy using serial sections and 3D reconstruction between rhesus monkeys and humans showed that the urogenital diaphragm, and therefore, the DTP neither existed in the rhesus monkey nor in the human male (Dorschner et al., 2001). However, in both, a urethral sphincter muscle exists, independent of the pelvic floor musculature (Ganzer et al., 2004). Kaye et al. (1997) indicated that the presence of the urogenital diaphragm, consisting of the external urethral sphincter muscle and DTP muscle, separated from the prostate gland by a superior aponeurosis, is a “myth.” The authors found that the striated external urethral sphincter surrounding the membranous urethra extends from the perineal membrane immediately above the bulb of the penis and bulbospongiosus muscle up to and onto the anterior aspect of the prostate (Kaye et al., 1997). Based on their topographical findings during radical prostatectomy, they suggested that the term urogenital diaphragm be no longer used (Kaye et al., 1997). Oelrich (1980) described a separate male urethral sphincter as striated muscle in contact with the urethra, from the base of the bladder to the perineal membrane (Oelrich, 1980). However, he did not confirm the concept of a urogenital diaphragm that the urethral sphincter spans in a transverse plane between the two ischiopubic rami, with superior and inferior fascial coverings (Oelrich, 1980). Also in females, a separate DTP muscle was not found. Sometimes it becomes synonymous with the transverse vaginal and transverse urethral muscles (Oelrich, 1983). Since Oelrich found no superior fascia of the so‐called “urogenital diaphragm” in females, which closes off a deep perineal compartment or forms the floor of the urogenital hiatus, he introduced the term perineal membrane (Oelrich, 1983; Stein & DeLancey, 2008).

Meanwhile, the urogenital diaphragm is no longer mentioned. According to the official nomenclature (Terminologica anatomica 2019), the perineal membrane, which used to be the lower fascia of the urogenital diaphragm, runs between the lower left rami and right pubis below the hiatus levator, dividing the perineal pouch. The superficial perineal pouch is inferior (caudal) while the deep perineal pouch is superior (cranial) to the perineal membrane. The striated erectile tissue muscles (bulbospongiosus and ischiocavernosus muscles) and sphincter muscles (sphincter ani externus muscle) are localized in the superficial perineal pouch. Within the deep perineal pouch, the sphincter urethrae in males and compressor urethrae in females, as well as fibromuscular structures, are found (Bolla et al., 2024). The deep perineal pouch also contains the DTP muscle. Together with the perineal membrane, it forms a functional unit as the urethra support mechanism (Stoker, 2009). A striated DTP muscle is predominantly present in males (Schmeiser & Putz, 2000; Strandring, 2016; Waschke et al., 2015). Macroscopical and histological studies on the other side identified a rather small DTP, with partly an unclear morphology, mostly without a sheet‐like structure, even in elderly women and men (Nakajima et al., 2007).

Meanwhile, a fairly common understanding in the current textbooks is that the DTP does not exist in females but may be present in males (Betschart et al., 2008; Kureel et al., 2011; Paulsen & Waschke, 2022; Schunke et al., 2022; Waschke et al., 2015). Other studies also doubt the existence of this muscle in males (Kaye et al., 1997; Oelrich, 1980). Therefore, we performed macroscopic, light microscopic, and immunofluorescent as well as stereological studies to establish the existence of a striated DTP muscle in females and males, and determine the composition of the DTP. We hypothesize the DTP as a definable striated muscle belly present in elderly males but not in elderly female donors used in the dissection course.

## 2 MATERIALS AND METHODS

All investigations were conducted on human donors, who had voluntarily donated their bodies for medical research and teaching. They had provided informed consent and made a bequest of their bodies to our institute, the Hannover Medical School (MHH), during their lifetime. The authors state that every effort was made to comply with all local and international ethical guidelines and laws pertaining to the use of human cadaveric donors in anatomical research (Iwanaga et al., 2022). The study was approved by the university's Ethics Committee (No. 11266_BO_K_2024).

This study included five female and five male donors. The mean ages at death for the females and males were 81.2 (67–91) and 82.8 (73–89) years, respectively. Pre‐existing conditions and causes of death are listed in Table 1. Donors were chosen randomly without any exclusion criteria.

**TABLE 1:** Pre‐existing conditions and causes of death.

No.	Sex	Age (years)	Diseases, cause of death
1	M	89	Prostate carcinoma, metastasized
2	M	87	Pneumonia, cardiac decompensation, apoplexy, heart failure, femoral neck fracture
3	M	78	Multi‐organ failure, cardiorenal syndrome, chronic renal insufficiency, swollen scrotum, mitral valve insufficiency, spinal canal stenosis
4	M	73	Pleural carcinoma, bronchial carcinoma, metastasized
5	M	87	Acute heart failure, COPD, hypertension, AF, prostate carcinoma
6	F	91	Unknown
7	F	79	Central dysregulation, multiple myocardial infarctions, coronary heart disease
8	F	84	Decompensated heart failure, hypertension
9	F	67	Unknown
10	F	85	Unknown

Abbreviations: AF, atrial fibrillation; COPD, chronic obstructive pulmonary disease.

The donors were embalmed by perfusion via the femoral artery with a solution containing 10 L ethanol (99%), 0.25 L glycerol, 0.75 L formalin (37%), and 0.25 L phenoxyethanol. Depending on the constitution, the perfusion volume varied between 10 and 15 L (approx. 20% of body weight). Afterwards, the bodies were stored in cuvettes (70% alcohol) for about 1 year to ensure the immersion fixation.

### 2.1 Dissection

**FIGURE 2:**
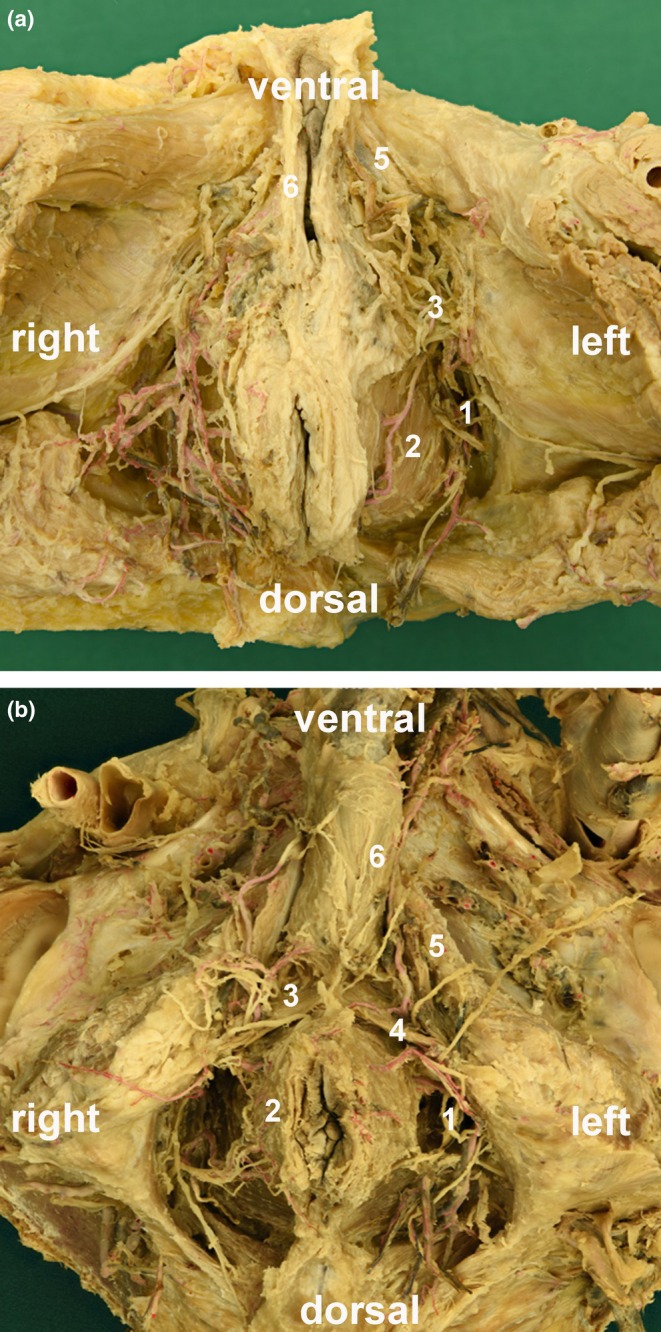
Perineal regions of (a) female and (b) male donors. Branches of the pudendal artery and nerve partly covering the deep perineal pouch. (1) Ischioanal fossa, (2) levator ani muscle, (3) perineal membrane/deep transverse perineal muscle covered by branches of the internal pudendal artery (inferior rectal artery), as well as branches of the pudendal nerve (perineal nerves and inferior rectal nerves), (4) superficial transverse perineal muscle (only visible in males), (5) ischiocavernous muscle, and (6) bulbospongiosus muscle.

The first step was to dissect the cutis and subcutis in the perineal region, so that the ischioanal fossa and structures of the urogenital region were exposed. The superficial perineal pouch muscles (ischiocavernosus and bulbospongiosus) were also exposed for orientation. However, the superficial transverse perineal muscle in the superficial perineal pouch was only seen occasionally, for example, in Figure 2. In both males and females, the deep perineal pouch was limited by the levator ani muscle (cranial) and perineal membrane (caudal). The lateral expansion spread out to both the pubic and ischial bones. The pudendal nerve and internal pudendal artery were removed, as well as other connective tissue and the external genitalia. However, all the transverse running tissues were preserved. After that, all the structures in the deep and superficial perineal pouches were excised. This allows the complete craniocaudal extent of the deep perineal pouch to be examined. The thickness of the dissected perineal structure was measured with a caliper. Subsequently, the excised trapezoidal tissue was divided into left and right portions and embedded.

### 2.2 Sampling and cutting

The dissected tissue block was additionally fixed with 1.5% paraformaldehyde/1.5% glutaraldehyde/0.15 M HEPES buffer or 4% paraformaldehyde/0.5% glutaraldehyde in 0.15 M HEPES buffer. After dehydration, the tissue was embedded with the deep perineal pouch at the bottom and the superficial perineal pouch facing the top of the block. After flat embedding in paraffin, serial cuts were made perpendicular to the surface of the dissected tissue block at 40 μm intervals, starting at the inferior part of the perineal membrane (from outside to inside), as long as the observed transversal fibers' courses were made. The 4 μm thick sections were stained with an azan solution containing Azokarmin G, acetic alcohol (96%), and phosphotungstic acid. The sections were then digitized with a scanner (AxioScan.Z1, Zeiss, Oberkochen, Germany) at an objective lens magnification of 20× for analysis with the newCAST stereology software (Visiopharm, Hørsholm, Denmark).

### 2.3 Stereology

To determine the histological composition of the tissue over the entire caudocranial extent, sections were randomly generated per sectional level. Approximately 30 and 50 fields of view were counted per section to obtain at least 100 to 200 counts of events per tissue component and donor.

Morphometrical measurements were carried out according to the guidelines for quantitative assessment structure (Tschanz et al., 2014; Weibel, 1979). The volume densities (*V*_Vstruct_) of defined structures, including the connective tissue (*V*_Vct_), smooth muscle cells (*V*_Vsmc_), and striated muscle fibers (*V*_Vsmf_), were determined using the connective tissue and muscle cells or fibers running transversally as the reference.

$$V_{V}\left(\text{struct}/\mathrm{ref}\right)=\mathrm{\Sigma P}\left(\text{struct}\right)/\mathrm{\Sigma P}\left(\mathrm{ref}\right).$$

Other structures, such as the bulbospongiosus muscle or blood vessels, were excluded from the analysis as well as from the reference (Figure 5a,b).

### 2.4 Immunohistochemistry (IHC)

IHC was performed using an antibody against smooth muscle actin. Sections of 4 μm thickness from the paraffin‐embedded tissue were mounted and deparaffinized with xylene. Subsequent sections were stained directly with azan (see above) or treated as follows. The sections were microwaved at 700 W for 14 min using a Dako retrieval buffer, pH = 6 (Dako, Glostrup, Denmark), to ensure antigen retrieval. Unspecific antigen binding was blocked with 5% donkey serum (Jackson ImmunoResearch Laboratories, Suffolk, United Kingdom), 1% bovine serum albumin (BSA), and 0.30% Triton‐X‐100 (Sigma‐Aldrich, Steinheim, Germany).

The sections were incubated with the primary antibody anti‐smooth muscle actin (diluted 1:100, Abcam, Cambridge, United Kingdom) diluted in PBS containing 1% BSA and 0.30% TX for 20 h. Next, the sections were washed and incubated with the secondary antibody, Alexa Fluor 546 donkey anti‐goat (diluted 1:1000, Thermo Fischer Scientific, MA, USA). Afterwards, the sections were washed again, incubated with DAPI nucleic acid stain (diluted 1:1000, Thermo Fischer Scientific), and embedded in fluorescence mounting medium and sealed with a cover slip. Digital images were taken with a slide scanner as mentioned above.

### 2.5 Statistical analysis

GraphPad Prism 6 (Dotmatics) was used as the software for creating the figures and for the statistical analysis. Data are reported as arithmetic mean ± standard deviation (SD), unless otherwise indicated. Stereological analyses were conducted, and statistical differences were considered as *p* < 0.05. We used the non‐parametric Mann–Whitney test because the data did not follow the Gaussian distribution.

## 3 RESULTS

**FIGURE 3:**
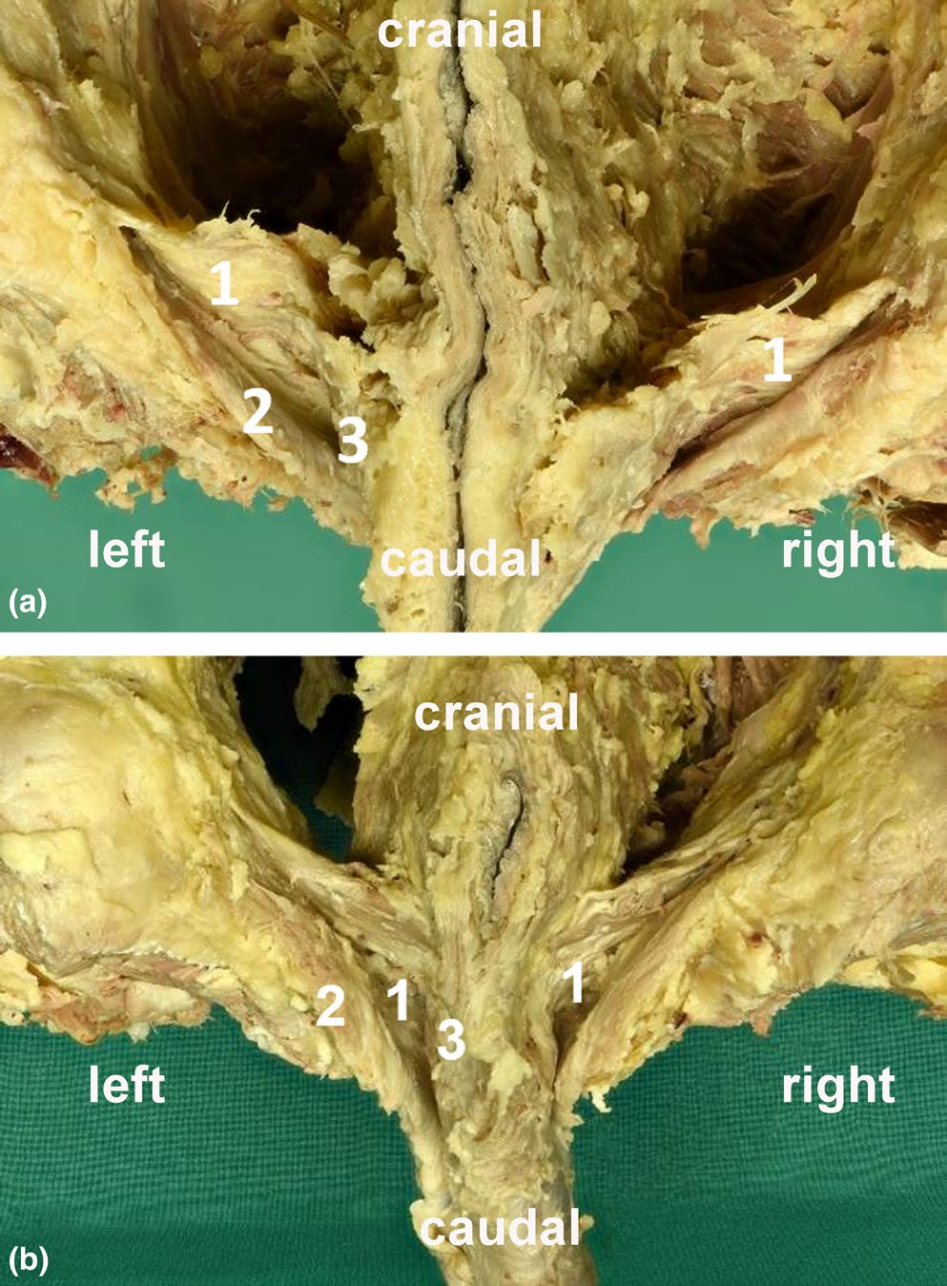
Posterior view of the perineal regions of (a) female and (b) male donors. After removing all nerves and vessels from the perineal membrane with the ischiocavernosus and bulbospongiosus muscle in the superficial perineal pouch are clearly visible. In the deep perineal pouch, no belly of transversally running muscle fibers located above the perineal membrane is visible in either females or in males. Around the urethral and vaginal openings, parts of the urogenital sphincter complex are visible. For histological studies only, the free part of the perineal membrane was used. (1) Deep transverse perineal muscle, (2) ischiocavernosus muscle, and (3) bulbospongiosus muscle.

**FIGURE 4:**
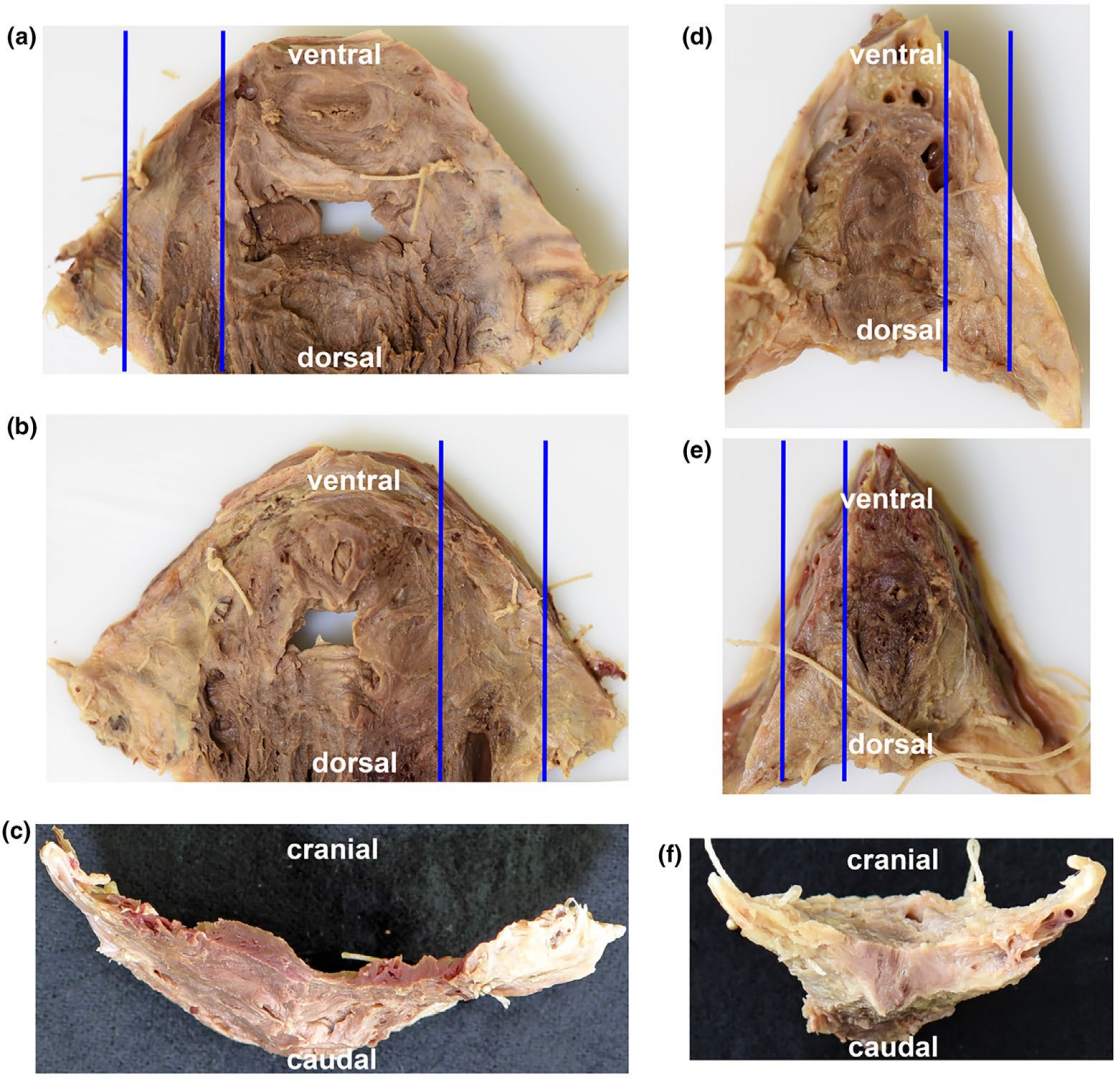
Excised whole tissue plate including the perineal membrane. (a–c) female; (d–f) male. (a) and (d) inner view; (b) and (e) outer views. The surrounding area of the urethral openings of the urogenital sphincter complex are visible. For histological studies only, the free part of the perineal membrane was used (limiting lines). (c, f) Frontal views of the whole plate.

Figure 2 shows the perineal membrane with the superiorly DTP as a transversally running plate in a female (Figure 2a) and male (Figure 2b). Both became more visible and prominent in male than in female donors. After removing the vessels and nerves, the contiguous connective tissue–muscular‐looking plate became clearly visible. A further independent, transverse muscle belly in the deep perineal pouch was not visible and could not be shown (Figure 3a,b). After the removal of the external genitalia and erectile tissue muscles, the continuous fibromuscular plate spreading between both pubic bones was dissected (Figure 4a–f). After excising the tissue stretched between the ischiopubic rami, size differences of the membrane became obvious between females (Figure 4a–c) and males (Figure 4d–f). The exemplary thickness of the plate (Figure 4c,f) was about 4.70 ± 1.3 mm in males (*n* = 7) and 3.75 ± 0.5 mm in females (*n* = 4). However, in general, the nature of the excised tissue plate varied. Sometimes, it was very thin and appeared like the connective tissue; at other times, it was thicker and had a more muscular appearance. The inferior portion is the perineal membrane; the superior portion is the DTP.

**FIGURE 5:**
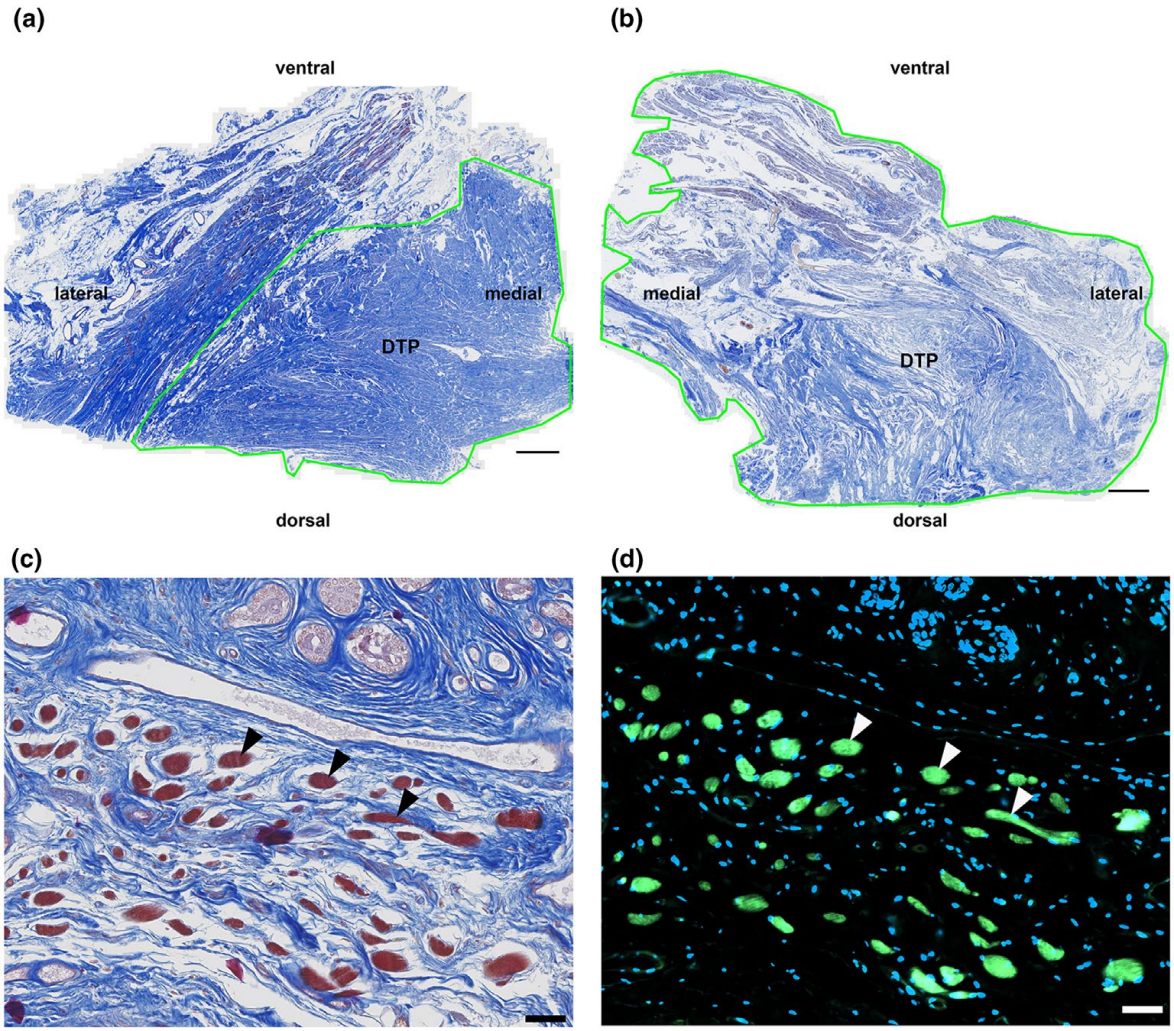
Histological section of (a) female (no. 7 in Table 1) and (b) male (no. 4 in Table) DTPs stained with azan. Transversally running collagen fibers (blue) are predominantly seen. Only limited muscle fibers (red) are visible. Green boxes indicate the counting frame for the stereological analysis. Scale bar: 0.2 Cm. (c, d) Male donor; age at death, 87 years (no. 5 in Table 1). Azan section (c) with subsequent immunofluorescence (d). Acidophilic structures, which were stereologically evaluated as smooth muscles (black arrows), are immunohistochemically positive for smooth muscle actin (white arrows). Merged channels with blue = DAPI and green = smooth muscle actin. Scale bar: 50 μm.

**FIGURE 6:**
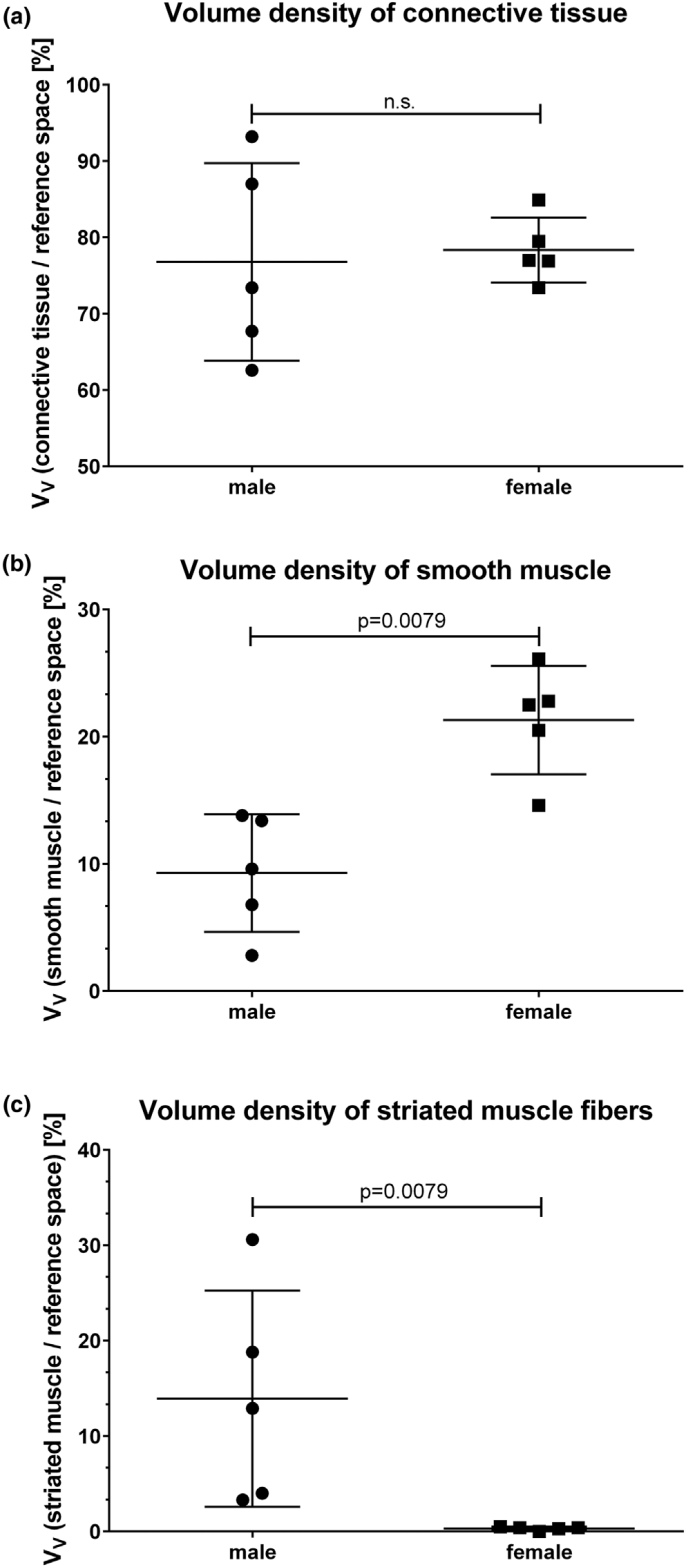
Stereological evaluation of the tissues of the deep perineal pouch, including the perineal membrane. (a) The volume density of the connective tissue is approximately 80%, comparable in both sexes. The volume density of the muscle cells is approximately 20% in both sexes; however, with a high standard deviation. (b) Volume density of smooth muscle cells is higher in females than in males. (c) Volume density of striated muscle fibers is significantly lower in females.

Serial sections of the tissue blocks contained transversally oriented connective tissues and definable smooth muscle cells (Figure 5a,b). The acidophilic tissue components in the azan staining (Figure 5c), which were stereologically evaluated as smooth muscle cells, were confirmed as such by detection of the smooth muscle actin using immunofluorescence (Figure 5d). The striated muscle fibers were negligible in females (Figure 6c). In males, the tissue blocks contained connective tissue and definable portions of smooth muscle cells and, in other planes, striated muscle fibers (Figure 6a–c).

The cranial part of the serial sections exhibited transversally running connective tissue and varying amounts of muscle tissue. Longitudinally oriented striated muscle fibers may be parts of the levator ani muscle (puborectal and pubococcygeus muscles). The caudal part of the serial sections apparently contained more connective tissue and longitudinally running striated muscle fibers, belonging to the urethral sphincter muscles as well as the bulbospongiosus muscle fibers bulging into the vaginal introitus on the medial side. To obtain quantitative information about the tissue distribution, stereological analytic methods were applied.

**FIGURE 7:**
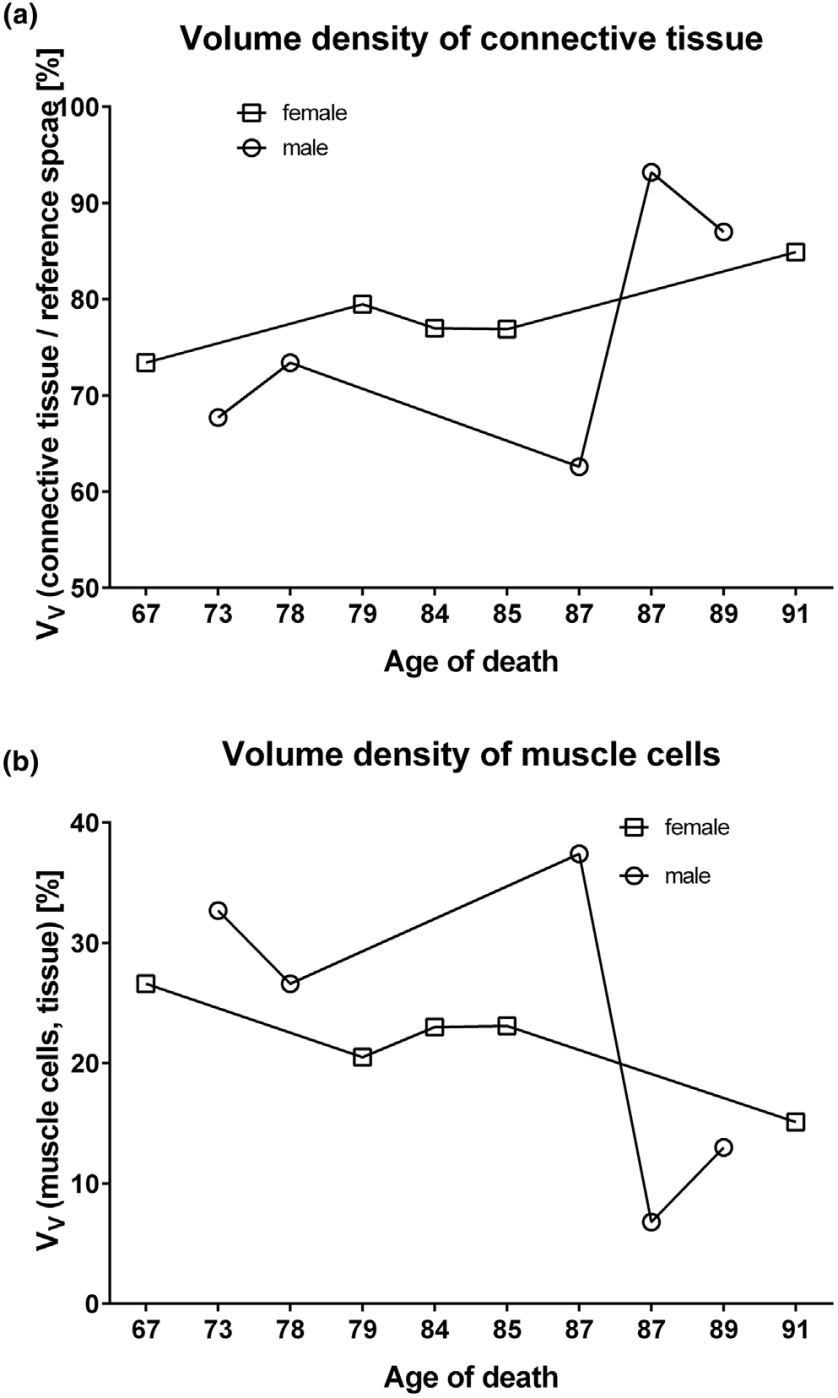
Stereological evaluation of the tissue of the deep perineal pouch, including the perineal membrane. (a) The volume density of the connective tissue increased during aging in both sexes. (b) The volume density of the muscle tissue decreased during aging in both sexes.

The main tissue compartment of the deep perineal pouch was the connective tissue (Figure 6a). The volume density of the connective tissue was nearly 80% and therefore significantly higher than the volume densities of muscles in both sexes, which were approximately 20% in both sexes. The portion of the smooth muscle cells was higher in females (Figure 6b). At nearly 15%, the fraction of the striated muscle fibers was significantly higher in males compared to that in females (Figure 6c). The ratio of muscle cells to connective tissue was higher in males than in females (43 ± 24 vs. 28 ± 8) without reaching significance because of the high standard deviation. With increasing age of the donors, we observed an increase in the volume density of the connective tissue, while that of the muscle decreased (Figure 7a,b). It should be noted that the values showed a great variance.

To summarize the results, the transversally running tissue plate, spreading between the ischiopubic rami, consists predominantly of connective tissue with a small amount of muscle tissue. In the female and male tissue plates, the volume densities of muscle cells were comparable. A defined muscle belly demarcating the connective tissue in the deep perineal pouch was not seen and could not be exposed.

## 4 DISCUSSION

During preparation of the pelvic floor, we exposed a nearly 5‐mm‐thick, tissue plate‐like structure between the ischiopubic bones and the external genital organs for both sexes. The distinct, continuous transversally running striated muscle in the deep perineal pouch was not observed. Microscopically, connective tissue was predominantly found in the serial cuts. Our findings support previous studies, which doubt the existence of a continuous, demarcated, and independent striated muscle (Dorschner et al., 1999, 2001; Ganzer et al., 2004; Kaye et al., 1997; Nakajima et al., 2007). Based on the stereological evaluation of the serial sections obtained from the different planes of the dissected tissue (perineal membrane and the deep perineal pouch tissue attached superiorly), the so‐called DTP consists predominantly of connective tissue, and only one fifth of the transversally running tissue consists of muscle. In males, 10% of the muscle tissue is striated muscle fibers. Compared with other muscles, the proportion of muscle cells or fibers in the DTP is significantly lower, as the following examples show. Hence, the volume density of smooth muscles in the myometrium of the uterus is 65% (Sweeney et al., 2013). Stereological studies of the anterior vaginal wall revealed that the volume density of smooth muscle cells amounts to 60%, and the volume density of connective tissue to 39% in younger females. In older females, the anterior vaginal wall contains 41% smooth muscle cells and 54% connective tissue (Babinski et al., 2022). Morphometrical analysis of muscle fibers in the human skeletal psoas major muscle showed a decrease in the fraction of striated muscle fibers with aging independent of sex (75% in humans with 30–49 years of age, and 66% in humans 70 years and older) (Antić et al., 2015).

The striated muscle fibers most likely belong to parts of the urethra support mechanism (external urethral sphincter or urethral rhabdosphincter), as these muscles are in part closely associated with the DTP, as has been shown histologically (Hinata et al., 2014; Nakajima et al., 2007; Zhai et al., 2011). Furthermore, it was demonstrated in females using magnetic resonance imaging (MRI) that the ventral perineal membrane, which is equal to the urogenital diaphragm, interconnects with the compressor urethrae, vestibular bulb, and levator ani (Brandon et al., 2009). The high variance suggests that the muscle fibers of the urethral sphincters are only partially stretched transversely toward the ischiopubic bones and probably decrease with age. Based on the literature, we also noticed that the term for the tissue in the deep perineal pouch varied partly. Sometimes, the term perineal membrane refers to a trilaminar musculofascial structure (Stein & DeLancey, 2008) or fascial structure (Hinata et al., 2014; Kato et al., 2008; Muro & Akita, 2023) including the DTP (Wu et al., 2018). The term urogenital diaphragm is also often used (Betschart et al., 2008; Kaye et al., 1997; Nakajima et al., 2007). Some publications distinguished the perineal membrane from the DTP in the deep perineal pouch (Hinata et al., 2014; Muro et al., 2025; Nakajima et al., 2007).

Our quantitative findings mostly confirm the qualitative findings of earlier studies conducted predominantly on females, rarely on both sexes. When the perineal region of young females (0–37 years old) was investigated using serial histological sections of pelvis specimens and serial macroscopic cross‐sections of the entire pelvis of females aged 28–56 years, the dorsal and ventral regions of the perineal membrane were revealed (Stein & DeLancey, 2008). Further description of both portions is consistent with our findings. The dorsal portion is described as a bilateral transverse fibrous sheet attached to the lateral wall of the vagina and the ischiopubic bone, without any striated muscle. It is bound caudally by the fat of the ischioanal fossa and below by perineal structures, such as the vestibular bulb and clitoral crus, with their enfolding bulbospongiosus and ischiocavernosus muscles. The dorsal edge of the perineal membrane was limited by the superficial transverse muscle in the superficial perineal pouch when present. The ventral portion of the membrane contains, as part of a solid three‐dimensional tissue mass, several structures such as the paraurethral and paravaginal connective tissues. Additionally, this part is closely associated with the compressor urethrae and urethrovaginal sphincter muscles of the distal urethra. The levator ani muscles are attached to the cranial surface of the perineal membrane complex (Oelrich, 1983; Stein & DeLancey, 2008). Furthermore, MRI studies suspected a striated DTP (Brandon et al., 2009; Dorschner et al., 1999; Stoker, 2009). They defined the perineal membrane in females—also equated with the trilayered structure of the urogenital diaphragm—as a musculofascial unilayer structure. Muscle fibers in the deep perineal pouch previously assigned to the urogenital diaphragm most likely form part of the urethra support mechanism (compressor urethrae and urethrovaginalis) as part of the external urethral sphincter muscle (Stoker, 2009). Although the tissue plate is surrounded inferiorly by the perineal membrane and superiorly by the levator ani, we excluded these muscle fibers because we removed them macroscopically.

In a recent review, the DTP was characterized as a plate‐like structure consisting of smooth muscle cells oriented transversally and densely packed. The perineal membrane was defined as a fibrous structure to which the DTP is partially attached (Muro & Akita, 2023). Earlier publications suggested based on histological findings that the presence of smooth muscle cells in the DTP is as a plate‐like structure (Muro et al., 2018, 2021; Wu et al., 2018). In a more recent paper, using serial sections and a 3D reconstruction, transversely oriented densely smooth muscle cells belonging to the DTP and obtained from the deep perineal pouch were seen under the light microscope (Muro et al., 2025). However, all these studies describing the DPT predominantly as a dense smooth muscle plate relied on non‐quantitative data. Using stereological methods (Hsia et al., 2010), we could show for the first time that the DTP consists mainly of connective tissue. Our data are in accordance with an earlier histomorphological study on elderly female donors. In that previous study, biopsies taken from the urogenital diaphragm were investigated morphometrically to examine the cross‐sectional area of the different biopsy tissues (Betschart et al., 2008). Comparable to our results, they found the highest cross‐sectional area for the connective tissue (approximately 26 mm^2^ [80%]), followed by smooth muscle (approximately 5 mm^2^ [15%]), and striated muscle (approximately 2 mm^2^ [6%]). The measured thickness of the urogenital diaphragm was about 5.5 mm (range 1.6 to 10.5 mm) (Betschart et al., 2008). These data are within the measurement range for our females. However, they did not report data on the male DTP.

Because we were still able to determine some sex‐based differences in perineal membrane tissue composition and also a decreased volume density of transverse muscle fibers with increasing age; therefore, the presence of a uniform thick muscle plate cannot be completely ruled out in younger individuals.

Regarding our measurements, we suggest that only a weak smooth muscle plate development occurs in older donors. Possible disappearance of the striated levator ani and urethral muscles during aging was also discussed in other publications (Betschart et al., 2008; Jundt et al., 2005). A decrease in the fraction of muscle cells/fibers with aging was also described in the anterior vaginal wall (Babinski et al., 2022) as well as in the psoas muscle (Antić et al., 2015).

However, the DTP was still described in a recent review (Muro & Akita, 2023). This description of the relationship between DTP and perineal membrane may support our findings regarding a decrease in the muscle body during aging in both sexes. The DTP connects various skeletal muscles of the pelvic floor, creating a coordinated muscle structure to transmit forces. The perineal membrane containing the DTP serves as structural support and maintains the integrity of the deep perineal pouch (Muro & Akita, 2023). It is assumed that both contribute to providing stability and functionality of the pelvic floor (Muro & Akita, 2023). Therefore, the presence of relatively striated muscle fibers belonging to the urethral sphincters and partially extending into the DTP is understandable, as the DTP is believed to play a key role in the dynamic coordination between smooth and skeletal muscles, which is crucial for the functionality and stability of the pelvic floor (Muro & Akita, 2023). In contrast to other studies, by describing the DTP in association with the urethral sphincter muscles in the pelvic floor using mostly histological gross sections of the frontal or transversal structures, this is the first study to examine the exposed DTP macroscopically and histologically using morphometrical methods.

## 5 CONCLUSION

We found neither in males nor in females a prominent striated transversally running muscle in the deep perineal pouch. In the literature, different terms such as urogenital diaphragm, perineal membrane, and DTP muscle were used, but are referring to the same tissue plate spreading between both ischiopubic bones, which separates the superficial from the deep perineal pouch. Depending on age and sex, the tissue plate contains relatively smooth muscle cells. Striated muscle fibers associated with the DTP are part of the urethral sphincter muscles, diverging in the DTP. The DTP consists of smooth muscle cells and connective tissue in both sexes. With aging, muscle cells decrease and connective tissue increases, leading to a small fibromuscular plate‐like structure superiorly of a fascial structure, determined as the perineal membrane.

## AUTHOR CONTRIBUTIONS

Morten Kampelmann contributed to concept/design, acquisition of data, data analysis/interpretation, and drafting of the manuscript. Carina Grindley contributed to the acquisition of data. Christian Mühlfeld contributed to data analysis/interpretation and editing of the manuscript. Andreas Schmiedl contributed to concept/design, acquisition of data, data analysis/interpretation, and drafting of the manuscript. All authors revised the manuscript and approved the final version of the article.

## CONFLICT OF INTEREST STATEMENT

The authors declare no conflicts of interest.

## Data Availability

The data that support the findings of this study are available from the corresponding author upon reasonable request.

## References

[joa70057-bib-0001] Antić, V.M. , Stefanović, N. , Jovanović, I. , Antić, M. , Milić, M. , Krstić, M. et al. (2015) Morphometric analysis of somatotropic cells of the adenohypophysis and muscle fibers of the psoas muscle in the process of aging in humans. Annals of Anatomy, 200, 44–53. 25769135 10.1016/j.aanat.2015.01.006

[joa70057-bib-0002] Babinski, M.D.S.D. , Pires, L.A.S. , Fonseca, J.A. , Manaia, J.H.M. & Babinski, M.A. (2022) Fibrous components of extracellular matrix and smooth muscle of the vaginal wall in young and postmenopausal women: stereological analysis. Tissue and Cell, 74, 101682. 34800880 10.1016/j.tice.2021.101682

[joa70057-bib-0003] Betschart, C. , Scheiner, D. , Maake, C. , Vich, M. , Slomianka, L. , Fink, D. et al. (2008) Histomorphological analysis of the urogenital diaphragm in elderly women: a cadaver study. International Urogynecology Journal and Pelvic Floor Dysfunction, 19, 1477–1481. 18575798 10.1007/s00192-008-0669-9

[joa70057-bib-0004] Bolla, S.R. , Hoare, B.S. & Varacallo, M. (2024) Anatomy, abdomen and pelvis: deep perineal space. In: StatPearls. Bin Faisal: StatPearls Publishing. 30855860

[joa70057-bib-0005] Brandon, C.J. , Lewicky‐Gaupp, C. , Larson, K.A. & DeLancey, J.O. (2009) Anatomy of the perineal membrane as seen in magnetic resonance images of nulliparous women. American Journal of Obstetrics and Gynecology, 200, 583–586. 10.1016/j.ajog.2009.03.004 PMC2696929 19375575

[joa70057-bib-0006] Dorschner, W. , Biesold, M. , Schmidt, F. & Stolzenburg, J.U. (1999) The dispute about the external sphincter and the urogenital diaphragm. The Journal of Urology, 162, 1942–1945. 10569543 10.1016/S0022-5347(05)68074-3

[joa70057-bib-0007] Dorschner, W. , Stolzenburg, J.‐U. & Neuhaus, J. (2001) Structure and function of the bladder neck. Advances in Anatomy, Embryology, and Cell Biology, 159, 1–109. 10.1007/978-3-642-56879-4 11417142

[joa70057-bib-0008] Fritsch, H. , Lienemann, A. , Brenner, E. & Ludwikowski, B. (2004) Clinical anatomy of the pelvic floor. Advances in Anatomy, Embryology, and Cell Biology, 175, 1–64. 10.1007/978-3-642-18548-9 15152384

[joa70057-bib-0009] Ganzer, R. , Kohler, D. , Neuhaus, J. , Dorschner, W. & Stolzenburg, J.U. (2004) Is the rhesus monkey (Macaca mulatta) comparable to humans? Histomorphology of the sphincteric musculature of the lower urinary tract including 3D‐reconstruction. Anatomia, Histologia, Embryologia, 33, 355–361. 15540995 10.1111/j.1439-0264.2004.00576.x

[joa70057-bib-0010] Henle, J. (1873) Handbuch der Eingeweidelehre des Menschen. Braunschweig: Friedrich Vieweg.

[joa70057-bib-0011] Hinata, N. , Hieda, K. , Sasaki, H. , Kurokawa, T. , Miyake, H. , Fujisawa, M. et al. (2014) Nerves and fasciae in and around the paracolpium or paravaginal tissue: an immunohistochemical study using elderly donated cadavers. Anatomy & Cell Biology, 47, 44–54. 24693482 10.5115/acb.2014.47.1.44 PMC3968266

[joa70057-bib-0012] Hsia, C.C. , Hyde, D.M. , Ochs, M. & Weibel, E.R. (2010) An official research policy statement of the American Thoracic Society/European Respiratory Society: standards for quantitative assessment of lung structure. American Journal of Respiratory and Critical Care Medicine, 181, 394–418. 20130146 10.1164/rccm.200809-1522ST PMC5455840

[joa70057-bib-0040] Iwanaga, J. , Singh, V. , Takeda, S. , Ogeng'o, J. , Kim, H. , Moryś, J. et al. (2022) Standardized statement for the ethical use of human cadaveric tissues in anatomy research papers: recommendations from *Anatomical Journal* Editors‐in‐Chief. Clinical Anatomy, 35(4), 526–528. 35218594 10.1002/ca.23849

[joa70057-bib-0013] Jundt, K. , Kiening, M. , Fischer, P. , Bergauer, F. , Rauch, E. , Janni, W. et al. (2005) Is the histomorphological concept of the female pelvic floor and its changes due to age and vaginal delivery correct? Neurourology and Urodynamics, 24, 44–50. 15573382 10.1002/nau.20080

[joa70057-bib-0014] Kato, M. , Matsubara, A. , Murakami, G. , Abe, S. , Ide, Y. , Sato, I. et al. (2008) Female perineal membrane: a study using pelvic floor semiserial sections from elderly nulliparous and multiparous women. International Urogynecology Journal and Pelvic Floor Dysfunction, 19, 1663–1670. 18688559 10.1007/s00192-008-0701-0

[joa70057-bib-0015] Kaye, K.W. , Milne, N. , Creed, K. & van der Werf, B. (1997) The ‘urogenital diaphragm’, external urethral sphincter and radical prostatectomy. Australian and New Zealand Journal of Surgery, 67, 40–44. 9033375

[joa70057-bib-0016] Krosigk, E. (2012) Die anatomie des menschlichen beckens. Saarbrücken: VDM Verlag Dr. Müller.

[joa70057-bib-0017] Kureel, S.N. , Gupta, A. & Gupta, R.K. (2011) Surgical anatomy of urogenital diaphragm and course of its vessels in exstrophy‐epispadias. Urology, 78, 159–163. 21256552 10.1016/j.urology.2010.11.026

[joa70057-bib-0018] Mirilas, P. & Skandalakis, J.E. (2004) Urogenital diaphragm: an erroneous concept casting its shadow over the sphincter urethrae and deep perineal space. Journal of the American College of Surgeons, 198, 279–290. 14759786 10.1016/j.jamcollsurg.2003.07.022

[joa70057-bib-0019] Muro, S. & Akita, K. (2023) Pelvic floor and perineal muscles: a dynamic coordination between skeletal and smooth muscles on pelvic floor stabilization. Anatomical Science International, 98, 407–425. 36961619 10.1007/s12565-023-00717-7 PMC10256674

[joa70057-bib-0020] Muro, S. , Chang, L. , Tharnmanularp, S. , Nimura, A. , Churei, H. & Akita, K. (2025) Presence of smooth muscle continuous with the rectal and vaginal walls in the deep perineal space prompts reconsideration of the deep transverse perineal muscle. Scientific Reports, 15, 23730. 40610722 10.1038/s41598-025-09585-9 PMC12229694

[joa70057-bib-0021] Muro, S. , Tsukada, Y. , Harada, M. , Ito, M. & Akita, K. (2018) Spatial distribution of smooth muscle tissue in the male pelvic floor with special reference to the lateral extent of the rectourethralis muscle: application to prostatectomy and proctectomy. Clinical Anatomy, 31, 1167–1176. 30113089 10.1002/ca.23254

[joa70057-bib-0022] Muro, S. , Tsukada, Y. , Ito, M. & Akita, K. (2021) The series of smooth muscle structures in the pelvic floors of men: dynamic coordination of smooth and skeletal muscles. Clinical Anatomy, 34, 272–282. 33347645 10.1002/ca.23713 PMC7898478

[joa70057-bib-0023] Nakajima, F. , Takenaka, A. , Uchiyama, E. , Hata, F. , Suzuki, D. & Murakami, G. (2007) Macroscopic and histotopographic study of the deep transverse perineal muscle (musculus transversus perinei profundus) in elderly Japanese. Annals of Anatomy, 189, 65–74. 17319611 10.1016/j.aanat.2006.06.014

[joa70057-bib-0024] Oelrich, T.M. (1980) The urethral sphincter muscle in the male. The American Journal of Anatomy, 158, 229–246. 7416058 10.1002/aja.1001580211

[joa70057-bib-0025] Oelrich, T.M. (1983) The striated urogenital sphincter muscle in the female. The Anatomical Record, 205, 223–232. 6846873 10.1002/ar.1092050213

[joa70057-bib-0026] Paulsen, F. & Waschke, J. (2022) Sobotta, Atlas der Anatomie. Amsterdam: Elsevier.

[joa70057-bib-0027] Roch, M. , Gaudreault, N. , Cyr, M.P. , Venne, G. , Bureau, N.J. & Morin, M. (2021) The female pelvic floor fascia anatomy: a systematic search and review. Life (Basel), 11, 900. 34575049 10.3390/life11090900 PMC8467746

[joa70057-bib-0028] Schmeiser, G. & Putz, R. (2000) The anatomy and function of the pelvic floor. Radiologe, 40, 429–436. 10890037 10.1007/s001170050692

[joa70057-bib-0029] Schunke, M. , Schulte, E. & Schumacher, U. (2022) Prometheus, lernatlas der anatomie [Allgemeine anatomie des bewegungsapparates]. Stuttgart: Thieme.

[joa70057-bib-0030] Stein, T.A. & DeLancey, J.O. (2008) Structure of the perineal membrane in females: gross and microscopic anatomy. Obstetrics and Gynecology, 111, 686–693. 18310372 10.1097/AOG.0b013e318163a9a5 PMC2775042

[joa70057-bib-0031] Stoker, J. (2009) Anorectal and pelvic floor anatomy. Best Practice & Research. Clinical Gastroenterology, 23, 463–475. 19647683 10.1016/j.bpg.2009.04.008

[joa70057-bib-0032] Strandring, S. (2016) Gray's anatomy: the anatomical basis of clinical practice. Anatomy series, Vol. 41. New York: Elsevier Limited.

[joa70057-bib-0033] Sweeney, E.M. , Crankshaw, D.J. , O'Brien, Y. , Dockery, P. & Morrison, J.J. (2013) Stereology of human myometrium in pregnancy: influence of maternal body mass index and age. American Journal of Obstetrics and Gynecology, 208(4), 324.e1–324.e6. 10.1016/j.ajog.2013.01.019 23333540

[joa70057-bib-0034] Thiele, J. , Dorschner, W. , Schmidt, F. , Stolzenburg, J.U. & Biesold, M. (1997) Nuclear magnetic resonance tomography examinations of the urogenital diaphragm in comparison with corresponding histomorphologic findings (the controversy concerning the musculus transversus perinei profundus). Aktuelle Radiologie, 7, 45–49. 9138523

[joa70057-bib-0035] Tschanz, S. , Schneider, J.P. & Knudsen, L. (2014) Design‐based stereology: planning, volumetry and sampling are crucial steps for a successful study. Annals of Anatomy, 196(1), 3–11. 23769130 10.1016/j.aanat.2013.04.011

[joa70057-bib-0036] Waschke, J. , Böckers, T.M. & Paulsen, F. (2015) Anatomie. München: Elsevier.

[joa70057-bib-0037] Weibel, E.R. (1979) Stereological methods. Practical methods for biological morphometry. New York: Academic Press.

[joa70057-bib-0038] Wu, Y. , Dabhoiwala, N.F. , Hagoort, J. , Hikspoors, J.P.J.M. , Tan, L.W. , Mommen, G. et al. (2018) Architecture of structures in the urogenital triangle of young adult males; comparison with females. Journal of Anatomy, 233, 447–459. 30051458 10.1111/joa.12864 PMC6131961

[joa70057-bib-0039] Zhai, L.D. , Liu, J. , Li, Y.S. , Ma, Q.T. & Yin, P. (2011) The male rectourethralis and deep transverse perineal muscles and their relationship to adjacent structures examined with successive slices of celloidin‐embedded pelvic viscera. European Urology, 59, 415–421. 21144644 10.1016/j.eururo.2010.11.030

